# Policymaker Perspectives on the Role of Health Systems in Sustainable Hepatitis C Point‐Of‐Care Testing in Australia

**DOI:** 10.1111/jvh.70080

**Published:** 2025-09-12

**Authors:** Anna Conway, Jason Grebely, Carla Treloar, Susan Matthews, Lise Lafferty, Natalie Taylor, Guillaume Fontaine, Alison D. Marshall

**Affiliations:** ^1^ The Kirby Institute University of New South Wales Sydney Australia; ^2^ Centre for Social Research in Health University of New South Wales Sydney Australia; ^3^ International Centre for Point‐Of‐Care Testing, Flinders Health and Medical Research Institute Flinders University Adelaide Australia; ^4^ School of Population Health University of New South Wales Sydney Australia; ^5^ Centre for Clinical Epidemiology Lady Davis Institute for Medical Research Montreal Canada; ^6^ Faculty of Medicine and Health Sciences McGill University Montreal Canada; ^7^ Centre for Implementation Research Ottawa Hospital Research Institute Ottawa Canada

## Abstract

Point‐of‐care testing for hepatitis C virus (HCV) offers multiple benefits to key populations and healthcare providers, but it has not achieved widespread implementation. This analysis investigates the impact of the health system on the sustainability of point‐of‐care HCV testing in Australia. Between September 2023 and January 2024, in‐depth, semi‐structured interviews were conducted with people involved in HCV policymaking in Australia. Data were coded using WHO's Health System Building Blocks framework (i.e., Health Workforce, Health System Financing, Medical Technologies, Leadership and Governance). Thematic analysis examined how the health system supports and hinders the long‐term sustainability of HCV point‐of‐care testing. There were 29 participants working in seven Australian jurisdictions or nationally: 13 from departments of health, six from community‐led organisations, five from local health services, and five from pathology. The analysis demonstrates the interrelations between Building Blocks, but governance was consistently foregrounded across each theme. For Health Workforce, the community approach to models of care in Australia bolstered support for HCV testing outside of traditional healthcare settings. For Health System Financing, sustainability was threatened by a lack of long‐term funding mechanisms for point‐of‐care testing. For Leadership and Governance, state and national HCV elimination targets were seen as important to drive point‐of‐care testing at the local level, especially when they were reflected in services' key performance indicators. Integration into existing health system structures, sustainable funding mechanisms, and strengthened governance frameworks are needed to sustain HCV point‐of‐care testing in Australia. Study findings are critical to inform a long‐term testing strategy in Australia and internationally.

## 1 Introduction

Therapies which can cure more than 95% of people with hepatitis C virus (HCV) are one of the greatest medical advances in decades, reducing the risk of hepatocellular carcinoma, liver decompensation and overall mortality [1]. Rates of new HCV infections are declining in Australia [2], but HCV testing may have plateaued [3], threatening progress towards WHO elimination targets which aim to reduce new HCV infections among people who inject drugs to 2% per year by 2030 [4]. Efforts to increase the uptake of HCV testing are hampered by diagnostic pathways which require multiple visits by patients, often resulting in frequent loss to follow‐up which is amplified in key populations such as people who inject drugs [5].

Point‐of‐care testing for HCV has been identified as a key strategy to reduce gaps in the care cascade by simplifying diagnostic pathways [6]. Point‐of‐care testing reduces the number of visits required for a diagnosis, reducing loss to follow‐up and supporting single‐visit testing, diagnosis and treatment. HCV diagnostic tests either detect antibodies, indicating past or current infection, or RNA, which indicates a current infection. Point‐of‐care antibody testing has been shown to improve linkage to care and treatment uptake [7], while point‐of‐care RNA testing facilitates single visit diagnostic testing [8, 9] and treatment uptake [9, 10, 11]. Dried blood spot testing (where blood is collected on a Guthrie card and transported to the laboratory for testing) allows sample collection to be performed in non‐medical settings, increasing the reach of testing [12] and improving antibody testing uptake among people who inject drugs [13]. Dried blood spot testing and point‐of‐care HCV testing use fingerstick blood samples, reducing the need for venepuncture in people with poor venous access, such as people who inject drugs, who have a high burden of HCV [14]. The WHO has published guidance recommending the use of point‐of‐care RNA tests to diagnose HCV infection [15].

Previous literature has defined criteria of an ideal diagnostic test to inform disease control strategies, strengthen health systems and improve patient outcomes and has described the trade‐offs between accuracy, accessibility and affordability [16]. Testing is delivered at different levels of the healthcare system (by pathologists, specialist clinicians, general practitioners and community health workers) and the level of delivery influences how the trade‐offs are managed. The local epidemic is also an important factor; in settings with high HCV prevalence, point‐of‐care HCV RNA testing may be most appropriate while in settings with lower HCV prevalence, point‐of‐care HCV antibody testing followed by point‐of‐care HCV RNA testing may be preferable [17]. Additionally, point‐of‐care testing has been found to be more cost‐effective per treatment initiation compared to standard of care when testing groups at risk of HCV [17].

Despite rapid advancements in HCV testing technologies, health systems can impede progress to HCV elimination [14, 18]. Applying existing definitions of sustainability [19], a HCV point‐of‐care testing programme should continue to be delivered after a defined period of time, evolving or adapting (sustained), and it should continue to produce benefits for individuals/systems (sustainable). To ensure the health system can support sustainable models of HCV point‐of‐care testing, there is a need to understand policymakers' views on implementation. This analysis investigates the impact of the health system on the sustainability of point‐of‐care HCV testing in Australia.

## 2 Methods

This qualitative descriptive study was based on semi‐structured interviews conducted with people involved in HCV policymaking in Australia. Participants were identified and recruited from the research team's professional networks within Australia. A list of potential participants was constructed to reflect a range of jurisdictions and organisation types. The potential participants were invited to participate via email. Inclusion criteria for participants taking part in this study were being involved in HCV policymaking processes in Australia, being willing to have the interview recorded, and being over 18 years of age.

The study design evolved to account for the regulatory context in Australia that shapes the use of HCV point‐of‐care testing. At the time of writing, there were only two diagnostic HCV tests approved by the Therapeutic Goods Administration for use in Australia: one point‐of‐care test for HCV antibodies (INSTI HCV antibody test: bioLytical Laboratories, Richmond, Canada) and one point‐of‐care test for HCV RNA (Xpert HCV Viral Load Fingerstick test: Cepheid, Sunnyvale, USA). The GeneXpert machine is used in the Australian HCV Point‐of‐Care Testing Program [20], which has been implemented in 108 sites (> 400 testing locations) across the country and performed 35,554 tests between 2022 and 2024 and is ongoing. The Therapeutic Goods Administration categorises tests for HCV RNA as high risk (Class IV) in vitro diagnostic medical devices (IVD) [21], meaning they are required to meet more stringent training and quality regulations compared to tests in lower risk IVD classes. The Australian National Pathology Accreditation Advisory Council regulations for point‐of‐care testing performed by accredited pathology services require quality management strategies to reduce risk and patient harm including governance, record management, specimen collection processes, quality control and quality assurance processes, operator training, and ongoing competency and data communication and retention records [22]. Laboratory and testing regulations also govern how testing is financed. Currently, only specific HCV antibody and RNA tests performed by an accredited laboratory are included in the Medicare Benefits Schedule, meaning a rebate is available for the test [23]. As such, block funding from the federal government to the Australian HCV Point‐of‐Care Testing Program is the mechanism by which the delivery of HCV RNA point‐of‐care testing in Australia occurs at the moment.

The interview guide was developed to capture the macro‐level factors influencing implementation that emanate from the health system, clinics and state. Semi‐structured interviews were conducted by AC between September 2023 and January 2024 via videocall. Participants provided verbal consent prior to the interview. Participants who worked in community‐led organisations were compensated $AUD 50 for their time and other participants did not receive any financial reimbursement. Audio recordings of the interviews were transcribed verbatim by a transcriber working under a confidentiality agreement. Transcripts were deidentified and checked for accuracy by AC.

To understand the impact of the health system on the sustainability of HCV point‐of‐care testing, the WHO's Health System Building Blocks framework [24] was used for the initial coding. The WHO's Health System Building Blocks framework has previously been used to guide advocacy efforts on HCV elimination [14] and has been used in applied research to evaluate the interaction between health interventions and health systems [25, 26]. The six building blocks described in the framework are as follows:

- health workforce
- service delivery
- health information systems
- medical products, vaccines and technologies
- health system financing
- leadership and governance

**FIGURE 1:**
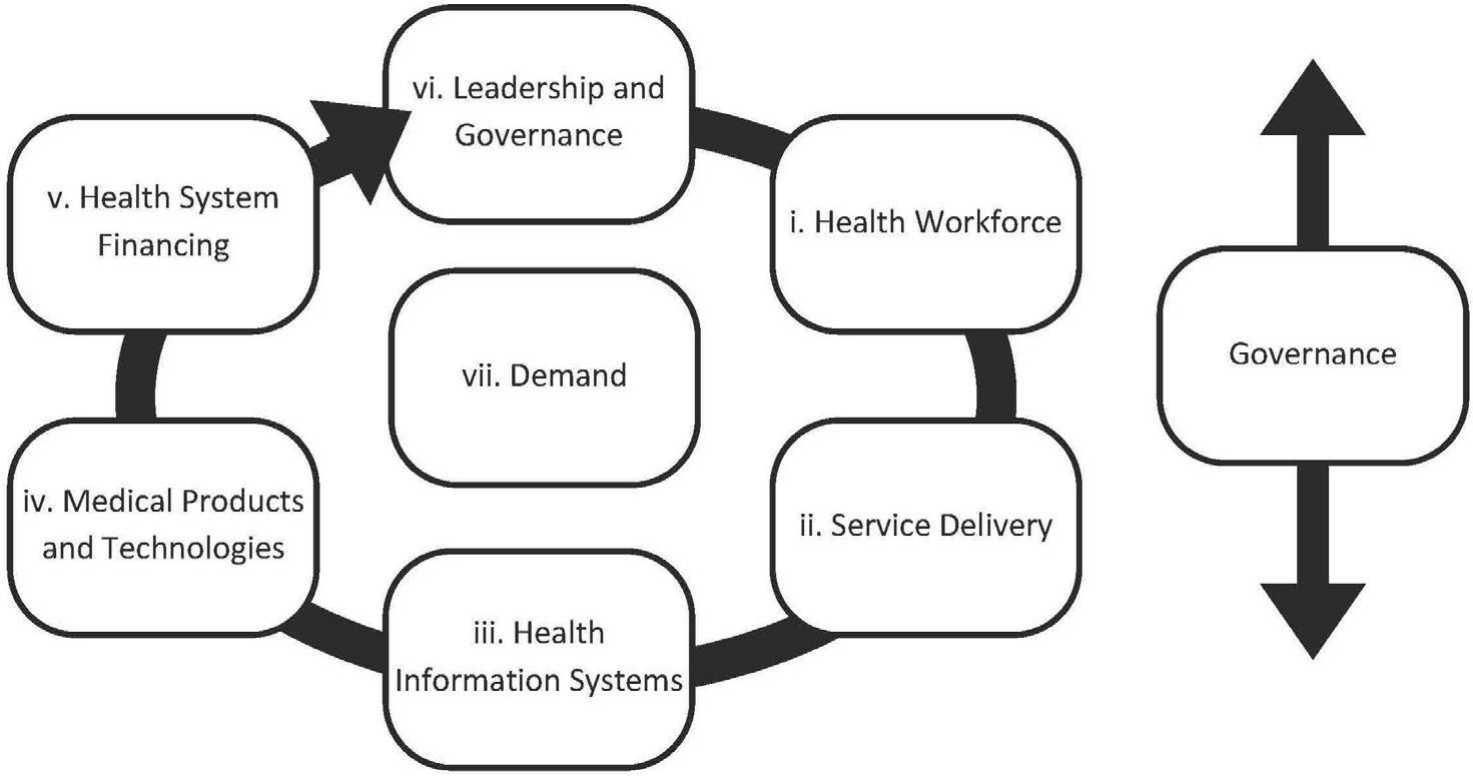
Conceptual framework for health systems to support a sustainable hepatitis C point‐of‐care testing strategy—adapted from the World Health Organisation Health System Building Blocks Framework.

Previous research studies have used this framework to investigate the performance of health systems and identify potential reforms [27, 28], and it has also been used to advocate for the adoption of HCV point‐of‐care testing globally [29]. There has also been recognition of the limitations of using such a framework to analyse systems impacts [30, 31], in part due to the Building Blocks' lack of focus on the demand side (factors that drive people's use of health services), no overarching health systems viewpoint, and no acknowledgment of interactions between the blocks. This analysis sought to adapt the framework and identify the interactions between Building Blocks to provide a richer understanding of the impact of the health system. As such, an adjusted framework that incorporates demand and a holistic health systems approach is used in this analysis to structure the findings. Interactions between the themes are explored in Figure 1 and the discussion.

Deductive coding was performed using the constructs from the WHO's Health System Building Blocks framework [24]. Inductive analysis was performed within each Building Block to situate participants' accounts within the health systems and HCV literature. The process allowed for the development of themes across the Building Blocks to inform a holistic viewpoint of the health system. The analysis is presented using the Building Blocks framework to draw together recommendations for real‐world application of the findings, and then to explore cross‐cutting themes in the discussion. AC led the coding, data coding and analysis processes, and the research team met regularly to review developing interpretations.

The study was approved by the UNSW Human Research Ethics Committee (HC230472).

## 3 Results

A total of 29 participants working in seven Australian jurisdictions or nationally were interviewed: 13 from state and federal departments of health, six from community‐led organisations (including drug user, disease‐specific and Aboriginal‐led community organisations), five from local health services, and five from pathology. There were 15 women, one non‐binary person, and 13 men interviewed. Participants had been employed in their current role for a median of 3 years (IQR 1–6 years) and had been working in the blood‐borne virus sector for a median of 15 years (IQR 6–22 years).

Figure 1 illustrates the components of the health system with regards to HCV point‐of‐care testing, visualising the interaction between the blocks. The analysis identified the reciprocal relationship between service delivery, health workforce, information systems, medical products and financing. The interviews highlight Demand as a preliminary block that is effectively a driving force shaping what the health system needs to do, while Governance cuts across the other blocks and is key to gaining a holistic, ‘whole system’ perspective (Figure 1). Table 1 summarises the barriers and facilitators that were identified in the interviews.

**TABLE 1:** Barriers and facilitators in the health system to achieve a sustainable hepatitis C point‐of‐care testing strategy in Australia.

	Barriers	Facilitators
Service delivery	A large and diverse group of test operators apply the quality assurance processes	Strong centralised coordination for testing implemented through research programmes
Health workforce	The success of local champions is dependent on staff turnover Large and diverse group of test operators complicates training Staff turnover complicates training	Skilled peer workforce in Australia Enthusiasm for HCV point‐of‐care testing among the people delivering it Local champions in sites and local health districts
Information	Fragmented information systems Lack of integration of GeneXpert platform into HCV notifications system Concerns about accountability of test results when integrated into HCV notifications system Double burden of work for local health districts reporting on point‐of‐care testing for surveillance systems and programmatic performance	Previous projects that provide point‐of‐care testing for STIs [32] have integrated the results of testing for other diseases using GeneXpert into surveillance systems
Medical products	There is no consistent algorithm to select the most appropriate testing modality (point‐of‐care antibody, dried blood spot testing, RNA testing) Xpert HCV Viral Load Fingerstick test still perceived as a screening test by some, despite Therapeutic Goods Administration approval Lack of Therapeutic Goods Administration approval for some testing modalities (e.g., some point‐of‐care antibody tests and dried blood spot testing) Maintenance of testing platforms, especially when testing is being delivered on outreach	Therapeutic Goods Administration approval of an HCV RNA test (Xpert HCV Viral Load Fingerstick test) allows for single visit diagnosis and treatment Availability of a suite of HCV point‐of‐care tests allows frontline workers to tailor testing to the needs of the person
Financing	No mechanisms to reimburse providers for HCV point‐of‐care testing Difficulty gaining approval from the Therapeutic Goods Administration for high‐performing testing technologies that do not have a strong commercial case Structural stigma may prevent community organisations from obtaining government funding to provide point‐of‐care testing and may prevent health services from contracting peers to deliver testing	
Leadership and governance	Governance of point‐of‐care testing platforms when used on outreach, outside of fixed sites	HCV elimination targets generate political will in support of HCV point‐of‐care testing HCV elimination targets provide parameters to drive testing at the local health districts and sites level Broad support for agile testing modalities, particularly in areas where HCV prevalence is low
Demand	Competing priorities (e.g., increasing prevalence of syphilis)	Engagement with affected populations in development of HCV strategies increases use and impact Sensitisation to point‐of‐care testing as a result of the COVID‐19 pandemic
Overarching, holistic health systems viewpoint	Stigma and discrimination experienced by people who inject drugs and people with HCV infection Criminalisation of drugs that creates barriers to care	Point‐of‐care testing as a tool to respond to stigma and discrimination

### 3.1 Health Workforce

A strong health workforce requires working ‘in ways that are responsive, fair and efficient to achieve the best health outcomes possible, given available resources and circumstances’ [24]. The values and capabilities of the existing workforce can present a challenge to implementing innovation. Governance can address issues around quality assurance, by ensuring staff have relevant skillsets and there is a protocol for responding to staff turnover. This may be insufficient for some professionals where a more complex cultural shift is required to deliver point‐of‐care testing.

Community organisations were recognised as a key partner to deliver services. Australia has a long history of organised drug user movements and the active involvement of people who inject drugs in the HIV and HCV response [33], contributing to a skilled peer workforce who can support HCV testing and treatment [34]. P27 explains how this context strengthens the health system.

And community organisations play a role in that implementation plan. We probably wouldn't be able to have a good success story without them and I notice this across a number of diseases. Australia is unique in its sort of community mindset or its community approach to a lot of models of care. P27 (Department of Health)

Although the professionalised community‐led organisations were highlighted as a key strength in the sustainability of the HCV point‐of‐care testing strategy, one participant saw a gap that may contribute to social equity in health. P24 (community‐led organisation) suggested that ‘we haven't really invested in establishing the peer workforce for Aboriginal and Torres Strait Islander people’, indicating the need for peer workers to be available to all key affected populations.

Participants suggested that the success of point‐of‐care testing was reliant upon members of the workforce valuing the intervention which, in turn, drove demand (See Section 3.7). Enthusiasm and support from staff at the local level were important to deliver new models of care. P7 reflects prior literature that identified champions as important to facilitate implementation at the site level and across sites [35, 36].

[Interviewer: Do you think there are characteristics common to those local health districts which used the testing platforms extensively?] Enthusiasm and a champion. I think you've got that one champion that sets each of the different sites up and that one person that sees it through also, to pass it from user to user. I think maybe, once the model matures a little bit more and people are confident with the set‐up and the structure … But I really think it takes a champion to be able to successfully set a model like that up. P7 (Department of Health)

Finally, the skills required to deliver point‐of‐care testing depended on the modality. For example, the training for dried blood spot sampling was less onerous than for becoming a GeneXpert operator and the former could be delivered in a ‘training the trainer’ model without scheduling with a central coordinator. A staff member in a local health district noted that regulatory requirements for training to use GeneXpert devices produced ‘another barrier because […] staff don't just stay at the service for the duration of the point‐of‐care testing project’ (P21). Training is required to comply with Therapeutic Goods Administration regulations but exacerbates the impact of high staff turnover on sustained point‐of‐care testing.

### 3.2 Service Delivery

Optimum service delivery involves delivering ‘effective, safe, quality personal and non‐personal health interventions to those that need them, when and where needed, with minimum waste of resources’ [24]. Participants consistently referred to the role of quality assurance processes as being key to delivering safe and quality testing with minimal patient harm.

In Australia, a number of point‐of‐care testing programmes are currently carried out by research institutes or within the context of research studies [20, 37, 38] where there is strong coordination to support service delivery, train and maintain operator competency and provide sustained technical support. Existing literature highlights the crucial role of research teams to support intervention uptake through their personal engagement and coordination support [39]. Coordination work in HCV point‐of‐care testing is varied but consists of tasks such as maintaining test devices, performing operator training, reviewing and actioning quality control and quality assurance test results, and providing clinical governance and IT support [20]. When considering integration into standard of care, P15 was concerned that a potential lack of centralised coordination in the future could be detrimental to quality of care.

In terms of scale‐up, it theoretically could get really fragmented if there's no regulations, if there's no coordination, if there's cowboys going out, doing things. You know, if they, if someone thinks they could make a quick buck out of it […] But there is, potentially, a lack of coordination if scale‐up is conducted in an uncontrolled or uncoordinated manner. P15 (Pathology)

For one pathologist, an important aspect of quality assurance was investment in ‘improving the quality of collection of samples at the site’ (P29). For another, training was insufficient to guarantee quality assurance processes would be followed. The potential for point‐of‐care testing to be delivered by trained lay people, including non‐clinical staff such as peer workers, contributed to concerns about maintaining quality in the testing processes. This quote from P16 indicates a lack of confidence in quality assurance processes when testing is being delivered by a range of staff.

So, capacity‐building and training are really not the issues, they're very simple to sort of get around. It's consistency of testing and it's adherence to processes and procedures that becomes the really difficult part because you've got a large, diverse group of people doing, what is nowadays, a highly sensitive and specific test. And if one person decides to wander from the protocol you can have some really significant impacts. P16 (Pathology)

Ensuring quality assurance training and processes are suitable for a range of staff was also a priority for community organisations, who emphasised equal importance for the safety of the test result and the cultural safety for the community being served. A participant from a community organisation felt that quality assurance trainings delivered by research institutes were necessary with regards to the ‘medical side of things’ (P23). At the same time, P23 felt that community organisations would be better placed to deliver training to prepare organisations to provide testing to key populations because they have expertise generated from lived experience.

### 3.3 Health Information Systems

‘A well‐functioning health information system is one that ensures the production, analysis, dissemination, and use of reliable and timely information on health determinants, health system performance, and health status’ [24]. Existing information systems need to respond and adapt to support the integration of health innovations. Participants highlighted how governance of health information systems needed to be strong to ensure accountability in maintenance of information, in information production, and in use of the information.

Fragmented information systems were repeatedly identified as an issue for sustainable HCV point‐of‐care testing. Primarily, the systems in most states did not support automatic upload of HCV RNA test results from the GeneXpert platform to public health systems for monitoring mandatory HCV notifications. Lack of integration across different systems makes it difficult to resolve this problem for HCV point‐of‐care testing alone. Previous point‐of‐care programmes in Australia have set a benchmark for coherent information systems to support testing for other notifiable infectious diseases. TTANGO, a randomised controlled trial that has evolved into a national programme [32], was referenced as a successful example where the results of chlamydia, gonorrhoea, and trichomonas point‐of‐care tests were integrated into surveillance systems.

So, [feeding results into surveillance systems is] occurring really well for the TTANGO point‐of‐care testing programme. We need to set up something similar, so an automated, electronic feed from GeneXpert into our surveillance system. Well, it should go into our public laboratory provider and then get fed electronically through to our surveillance system as a kind of single flow of data. P9 (Department of Health)

The integration of data from point‐of‐care testing into formal notification and surveillance systems was flagged as a quality concern. By integrating point‐of‐care diagnoses and laboratory information systems, one pathologist was concerned that, without a mechanism to indicate where the test was performed, it would create equivalency between the tests performed inside the laboratory and outside, thus making the laboratory accountable for tests that they had not performed. This extends the account of a pathologist in Section 3.2, who felt that quality assurance systems are insufficient to compensate for a broad range of people performing testing.

It's about governance, so, if there's a problem with this test, who do you call? Because if they call me and say, ‘This test is rubbish. What's going on?’ I can't say anything about it because I don't know who's done it or why, or I have no access to the nitty‐gritty. […] It was not totally straight‐forward in terms of just bunging the results into [pathology service portal]. P12 (Pathology)

There was high variance between the jurisdictions with regards to the integration of point‐of‐care testing into information systems. For some participants, it was not clear how or if point‐of‐care testing was being reported at the jurisdictional level. One department of health staff member said, ‘What's the testing rates? Who's doing what where? How's it being reported? So, yeah, I genuinely can't answer that’ (P10).

At the local level, there were limits to the use of notifications and state‐level surveillance systems in the measurement of programmatic performance. One service manager in local health noted that they had to report data to their department of health but also report to a local data collection system to ensure they were monitoring patient characteristics such as reinfection, cirrhosis, homelessness and country of birth to understand, ‘Are we reaching people that we think we need to reach?’ (P22, local health district). This policy ambiguity around reporting exists between and across jurisdictions that presents a challenge to national scale‐up.

### 3.4 Medical Products and Technologies

For successful implementation, medical products should have ‘assured quality, safety, efficacy and cost‐effectiveness, and scientifically sound and cost‐effective use’ [24]. Innovative technologies spark excitement and harness motivation for change but can be problematic when governance and regulatory systems cannot keep pace with technological advancements. As P16 (pathology) reported, the testing technology was unlikely to present a barrier to implementation; ‘I think technology is the least of your problems. Technology's almost always the least of your problems’. Participants understood the technology as inseparable from its health system context.

There were concerns around what ‘safety’ meant in the implementation of different modalities of testing. A participant from a department of health described point‐of‐care HCV antibody tests as ‘a bit of a tightrope’ (P3), stating that a negative result ‘could just be a presumptive negative, pending further testing’. This quote from P29 reflects the same sentiment but refers to point‐of‐care HCV RNA testing, despite the Xpert HCV Viral Load Fingerstick test being approved by the Australian Therapeutic Goods Administration to diagnose current HCV infection. Pathologists were consistently more wary about the implications of point‐of‐care testing results compared to other participants.

I guess there's still this notion that a point‐of‐care test done in a community is a tentative screening result. It's not a finalised, diagnostic, test result, right? So, in many cases, and HCV is no different, that the fact that you get a positive RNA in the community, you commence treatment, that patient will still need to have conventional blood tests done at some point. And I think, by and large, patients are still having repeat RNA testing. And, if they do that, they would presumably have an RNA test done in a lab at that follow‐up visit, when they're having their liver‐function tests and their chemistry tests done. So, the point‐of‐care is really the screening test. P29 (Pathology)

While some modalities of HCV point‐of‐care testing are approved by the Therapeutic Goods Administration (point‐of‐care HCV RNA testing using GeneXpert: Cepheid, Sunnyvale, USA and point‐of‐care HCV antibody, for example, INSTI: Biolytical Laboratories, Richmond, Canada), others are not (point‐of‐care HCV antibody test Bioline: Abbott Diagnostics, and dried blood spot sampling), presenting a barrier to sustainability because the range of appropriate tests are not available in all settings. For P14 (local health district), using an approved test means they ‘don't have to muck around’ and can give a ‘diagnosis on the spot’. P29 flags the difficulties of achieving approval for other tests while the Therapeutic Goods Administration categorises HCV in vitro diagnostics as ‘Class 4 – high public health risk’ because they are ‘intended to detect infectious agents capable of causing serious disease’ [21].

Oh, the main barrier [to scaling up use of dried blood spot sampling] is Therapeutic Goods Administration registration. Because HIV, HCV are highly regulated, class 4 in vitro diagnostic devices. They receive the highest degree of scrutiny in terms of their performance and quality. There's no product—serological or molecular—that allow for the testing of dried blood spot samples. P29 (Pathology)

### 3.5 Health System Financing

Strong financing ‘raises adequate funds for health, in ways that ensure people can use needed services, and are protected from financial catastrophe or impoverishment associated with having to pay for them and provides incentives for providers and users to be efficient’ [24]. Ensuring that innovations in health are compatible with a country's health system financing is a key step to achieving sustainability. The governance of approval for new medical technologies as well as the governance of funding were identified as key issues for financing.

The majority of HCV point‐of‐care testing in Australia is delivered by research institutes through block funding from the Commonwealth government, that presents a challenge for long‐term financing. Currently, the only infectious disease point‐of‐care tests that are reimbursed through Medicare (the publicly funded universal health care insurance scheme in Australia) is testing for sexually transmitted infections carried out in remote communities [40]. For one participant from a community organisation, facilitating a Medicare rebate for point‐of‐care testing was essential because ‘it supports services to have funding to then employ people to do [testing] but also signals to a service that this is a valued and important service that you can deliver’ (P24). Several participants said that financing HCV testing through research institutes was unsustainable.

We have a real problem in this country where any pathology that's done outside the laboratories does not attract a Medicare rebate […] The only mechanism available is either financing through research frameworks, which I'd say is questionable in terms of the ethics around that, because for some of these programs there isn't really a research question to be answered any more. They work. They're acceptable. They're scalable. There just isn't a funding mechanism in place. […] equity of access to testing for people in the community would benefit immensely from having some kind of funding arrangement so that these models can be sustainably implemented long term and attract a rebate. P9 (Department of Health)

Commercial viability was identified as a key issue for HCV testing modalities that still do not have approval of Therapeutic Goods Administration, such as dried blood spot sampling. One participant in a department of health saw this as an issue with the model of approval currently in place in Australia, that ‘assumes that a commercial entity will be the ones who take the process to the Therapeutic Goods Administration rather than a public good’ (P5). This was deemed especially problematic for dried blood spot sampling because ‘there's no commercial interest in it. It's a laboratory process, basically. It's not selling you a widget’ (P5). Another issue with commercial viability concerns the small population of Australia which means that ‘the attractiveness of Australia as a market for the manufacturers or the sponsors of these tests is not huge relative to the scale of other countries’ (P20).

One of the key benefits for HCV point‐of‐care testing is that it enables a range of non‐clinical providers, including peer workers, to deliver testing. Participants explained how structural stigma impacted financing mechanisms for peer workers. First, the ongoing criminalisation of drugs affects large numbers of people who use drugs, and health services may be unable to hire people with a criminal record. Second, as explained by P23, government funding for testing is more likely to go to research institutes than peer‐led organisations.

I think point‐of‐care testing needs to be scaled up, nationally. But the investment can't just go to [research institute], for example. It can go through [research institute], maybe, but I think it's good as well to reduce stigma and for the government to invest directly in drug‐user organisations, not only wanting to give money to researchers or other organisations because they look cleaner or because they're much more marketable and an easy sell. P23 (Community Organisation)

### 3.6 Leadership and Governance

Leadership and governance in a successful health system ‘involves ensuring strategic policy frameworks exist and are combined with effective oversight, coalition building, regulation, attention to system‐design and accountability’ [24]. Leadership was facilitated by HCV elimination targets and governance to allow delivery of point‐of‐care testing in non‐medical settings.

State and national targets to eliminate HCV by 2030 were seen as important to keep HCV on the political agenda. One staff member at a department of health said they leveraged targets to get buy‐in from agencies. They said point‐of‐care testing was ‘politically fantastic’ as progress towards elimination targets allowed key stakeholders to ‘wave their flag in the air and say’, ‘How great are we?’ (P9, department of health). In contrast, another participant from a state where progress had ‘stagnated’ (P4, department of health), felt it was necessary to wait for other states before implementing new strategies for elimination – ‘I need more evidence from other states that it works well and a bit of a cost‐benefit kind of thing of what they invested, what they got out of it’. (P4, department of health).

At the service level, WHO elimination targets [4] were enacted through key performance indicators that drove competition between services. No departments of health reported ‘penalising’ poor performing services, but some reported ranking services based on key performance indicators to generate ‘a little bit of shame and embarrassment if you're sitting at the bottom of the list’ (P1, department of health).

Sustainable HCV point‐of‐care testing is dependent on having a regulatory environment that supports testing outside of the laboratory and, more specifically, in non‐fixed sites such as outreach vans. One pathologist saw the laboratory as having a key role in future point‐of‐care testing programmes, noting the complexity of creating this regulatory environment with laboratory accreditation. P29 proposes resolving this by embedding quality assurance processes in the accreditation.

Lab accreditation, the way that it's set up is fixed to an address, alright? So, if you had [a service] with a GeneXpert in the van, and went off and did testing in the streets, how would the Pathology Accreditation Agency view that? Would the accreditation be given to the van or would it be given to the [service], or would it be given to the pathology provider who oversees? And my view is it's somewhere in between. I would think that the pathology accreditation agency would accredit the credited pathology provider who's taking responsibility for that machine and wherever it's being used and ensure that the people and its environment are contained and controlled in the best possible way and managed in a quality manner. P29 (Pathology)

### 3.7 Demand

A confluence of factors has driven demand for HCV point‐of‐care testing, including the ability of community organisations to deliver point‐of‐care testing, the ubiquity of point‐of‐care testing during COVID‐19, and advocacy among people from affected communities for progress towards HCV elimination. Participants noted that demand might be lower in settings/populations where HCV was not perceived to be a high priority, for example, communities with increasing prevalence of syphilis.

Progress towards elimination was identified as a potential threat to the sustained point‐of‐care testing because services were implementing testing strategies that had an increasingly lower yield of HCV diagnoses. For P5, the responsibility for adapting to the changing local epidemiology was transferred to the service in a call to use resources ‘efficiently’.

I mean we try and maintain a constant dialogue with the organisations who we fund, recognising that they're autonomous institutions as well. We're not there to direct and tell them what to do. You'd hope there's a certain level of maturity where they're also not going to chase up things that are giving you the ever‐diminishing returns, because the point is to try and use resources in the most efficient manner that we can. P5 (Department of Health)

The involvement of community organisations and people with lived experience of injecting drugs or HCV were consistently emphasised as important to an appropriate and sustainable HCV point‐of‐care testing strategy. Engagement with people with lived experience when designing state/national HCV testing strategies was noted as ‘really important, to make sure that what we're doing is actually practical on the ground and something that people will use’ (P18, department of health). Peer workers raised awareness around point‐of‐care testing, as well as ‘reducing stigma and discrimination’ in the services especially for people who do not often access healthcare (P4, department of health). In this way, the health workforce was intrinsic to driving demand among people who were at risk of HCV.

Participants reflected upon the contextual issues that might impact demand. The widespread use of point‐of‐care testing during the COVID‐19 pandemic was recognised as important in sensitising people (both providers and service users) to point‐of‐care testing. For one participant from a community organisation, the experience of COVID‐19 resulted in ‘more willingness to invest in point‐of‐care and really deep consideration of the types of contexts and situations where point‐of‐care has a lot of value’ (P24).

Depending on the state and population, other diseases such as syphilis were considered to be of greater concern than HCV and that had implications for resourcing and funding. One participant in a department of health felt that these shifts in focus were cyclical.

But, at the highest level, at this point in time, I don't see a lot of energy getting in behind hepatitis. So, at the moment, syphilis is their flavour of the month and everything's syphilis, syphilis, syphilis. And that will fade away, and then they'll find some other thing to focus on until they lose attention on that and move onto the next thing. P5 (Department of Health)

### 3.8 Overarching, Holistic Health Systems Viewpoint

Participants often took a holistic view of the health system, an aspect which is lacking in the standard WHO Building Blocks framework. Taking a whole systems view is necessary to evaluate sustainability and compensate for the limits of using the Building Blocks framework that have been addressed in prior literature [30]. There are limits to the extent to which issues in each Building Block can be resolved if the whole‐system issues are not first addressed.

Participants highlighted the importance of looking beyond sustained testing, which merely indicates a sustained quantity, to testing which is sustainable in that it continues to produce benefits for the population who is being tested. P11 (community organisation) expresses this saying that testing the same people every week causes testing to ‘lose its magic… its effectiveness in engagement’. P8 (department of health) reflected this in advocating for continuous monitoring to understand where point‐of‐care testing fits in the wider health system; ‘We want to know that we're testing the right people that, who wouldn't normally otherwise get tested through going through a GP service, for argument's sake’.

Siloed care was consistently highlighted as a weakness of the health system, creating barriers to accessing testing. P21 (local health district) said, ‘I think often it's not hard to reach community. It's a service that is hard to reach; not a community’. The potential of point‐of‐care testing technologies is limited by a lack of cohesion between sectors that can result in services being out of reach of the people who need them. While HCV strategies were viewed as integral to shaping leadership and governance, these strategies were also deemed critical to foster holistic health systems viewpoints by bringing different sectors ‘on board'.

Point‐of‐care testing was largely understood to be in line with the state and national strategies on HCV including the focus on addressing stigma. Unlike the issues of structural stigma in funding described in Section 3.5, point‐of‐care testing itself was considered to be a useful tool to respond to stigma experienced by people at risk of HCV. P26 (department of health) explained the connection with point‐of‐care testing saying, ‘I know we're a bit off‐topic but it's actually all part of the same mix, we're using [point‐of‐care testing] as the tool to help deal with some of the terrible stigma and discrimination issues that happen to people, they're linked’. Participants saw the potential of point‐of‐care testing as an innovative health product but also to facilitate health systems change and tailor care for people who inject drugs and other people disproportionately affected by HCV.

In Australia, people who inject drugs are one of the key populations at risk of HCV [41] and providing healthcare to them in a context of drug criminalisation generates multiple barriers to care. Past experiences shape how people engage with health services [42] and, although point‐of‐care testing may overcome some of those issues by moving testing out of traditional healthcare settings, the stigma produced by legal and social environments still persists.

I think the biggest [legal barrier which impact HCV point‐of‐care testing programmes] is the use of illicit drugs and people who might be criminalised as a result of their drug use maybe not wanting to interact with government services. And having like historical distrust of those services, which is completely understandable, putting yourself in those peoples' shoes. P18 (Department Of Health)

## 4 Discussion

The analysis demonstrates how the health system can challenge the sustainability of HCV point‐of‐care testing. Point‐of‐care diagnostics have the potential to increase the reach of HCV testing and simplify pathways to care but require strengthened health systems to actualise that potential. Using the WHO Health System Building Blocks, the analysis takes a holistic approach to explore how the key factors (blocks) interact and identify areas of the health system that can be strengthened to achieve sustainable HCV testing strategies. The findings from this study are critical in guiding health system considerations for other countries looking to implement national programmes for delivery of point‐of‐care testing for HCV infection and other infections.

Participants indicated different factors which affect the sustainment (continued use) of HCV point‐of‐care testing and sustainability (continued benefits). There are acute issues in health financing and governance which must be resolved to achieve sustainment, but the challenges for sustainability will likely evolve over time. Centralised coordination, a skilled and resilient workforce and engagement with affected populations are all factors which can support agile responses to changing needs. Due to the important role of both sustainment and sustainability, there is a need for constant monitoring of use and benefits of HCV point‐of‐care testing, the factors affecting both, and the relationship between the two, to ensure appropriate strategies are designed to support optimal use leading to maximum benefit.

The analysis identifies several health system‐level barriers and facilitators to achieving sustainable HCV point‐of‐care testing strategies that are summarised in Table 1. In spite of many facilitators in the Australian health system, there are several barriers that persist and align with those identified in global advocacy efforts [29]. Although the study was conducted in Australia, the Building Blocks framework allows the generation of findings to support sustainable HCV testing strategies in other contexts. While previous work has noted that community health was not considered in the Building Blocks framework [31], this analysis demonstrates how an adapted framework can acknowledge the key role of community health in health systems strengthening. The health workforce in Australia is strengthened by professionalised community‐led organisations [34, 43] and this, along with other context‐specific aspects of the health system, should be considered in applying the recommendations outside of Australia.

The analysis brings to the fore three aspects of governance that are interlinked: quality assurance, financing and information. The analysis affirms the importance of quality assurance processes [5, 6] and emphasises how those processes can generate confidence in new testing modalities in order to engage key stakeholders in the scale‐up of point‐of‐care testing. While point‐of‐care testing in community settings complements routine laboratory testing by extending the accessibility of pathology services to high‐risk populations, where the burden of disease is often high, robust quality management and quality assurance processes could alleviate the concerns of pathology staff to ensure they are engaged in the diversification of testing modalities. As previously stated, operator training and quality assurance processes for HCV RNA point‐of‐care testing form a mandatory part of the requirements from the Therapeutic Goods Administration for this Class IV IVD and therefore must be factored into planning for a sustainable point‐of‐care testing strategy, just as routine laboratory‐based testing relies on the quality assurance processes within laboratories. Quality assurance is multifaceted, beyond just quality control, and there is opportunity for multiple stakeholders to deliver quality management and training systems according to their expertise. There are implications for budgeting and oversight in planning quality assurance processes but ongoing programmes [20, 44] offer a template for how to address issues of patient safety associated with point‐of‐care testing.

Approval of a suite of high‐performing HCV diagnostics is an important step to a sustainable HCV point‐of‐care testing strategy [18, 29] and is entwined with long‐term financing. Testing modalities that have proven effective in research studies still face barriers to scale up because manufacturers do not see a business case for applying for their regulatory approval [45]. This is problematic in Australia where the smaller population makes it difficult to make a case for the commercial viability of new medical products and has been reported as a barrier to point‐of‐care testing for other diseases [45]. Once approved for use, the limited possibilities for point‐of‐care testing to be approved for Medicare reimbursement make it difficult to scale up HCV point‐of‐care testing and contribute to siloed care. As noted in prior literature [45], there is discordance between jurisdictional and national HCV strategies that promote point‐of‐care testing and the regulatory environment that makes point‐of‐care testing difficult to implement. Improved governance to support the approval of medical products that may not demonstrate immediate profitability and improved governance of financing mechanisms to respond to new medical products are important to signal the value of point‐of‐care testing and allow it to be integrated into standard care.

Fragmented information systems and the associated governance were identified as an issue in evaluating testing strategies and integrating point‐of‐care testing in national surveillance. Connectivity systems were developed to support the real‐time delivery of molecular point‐of‐care test results as part of the TTANGO project for STI testing in Australia [46], providing a template for similar systems for HCV RNA testing. For HCV, the concerns about the quality of testing translated into concerns about the governance of reporting and who would be accountable for the reported results. The different systems for clinic, jurisdictional and national reporting risk overburdening clinic staff, particularly when the reporting to surveillance systems does not serve for clinics to evaluate their own local performance. Point‐of‐care test results could enhance surveillance systems, but governance is needed to ensure key stakeholders are confident in the systems and that the work of reporting is not excessively burdensome.

To allow service delivery to adapt to different populations and settings, the health system must support flexibility. This is an important characteristic of community health services and often relies upon services delivered by peers, to reach populations who may not otherwise access health services. Flexibility supports innovation in the HCV response and allows staff to respond to the local epidemic, giving them ‘permission to go off‐piste’ [47]. Previous work on HCV treatment emphasises the importance of providers being able to offer ‘negotiated flexibility’ to improve access to care and improve trust in the HCV treatment system [48]. Flexibility is also needed in funding streams, to ensure they are not prejudicial to community‐based organisations and do not exclude the organisations who may be best placed to deliver testing. Increased investment in peers who can reach specific key populations, such as Aboriginal people, could support sustainable HCV point‐of‐care testing. A combination of governance and adaptive funding mechanisms could foster flexibility to support point‐of‐care testing.

The synergies between strong leadership and the Australian and jurisdictional HCV elimination targets [49, 50] were conducive to sustaining HCV point‐of‐care testing. The targets were seen by some to bolster advocacy efforts [45] and boost political will in support of point‐of‐care testing within departments of health. Conversely, progress towards elimination results in fewer people being identified with HCV, impacting the key performance indicators that are set for local health districts. Previous research has found a similar tension between finding people with HCV in a context of shrinking HCV prevalence, with participants asking ‘how long can we keep looking’? [47]. Contexts where HCV micro‐elimination has been achieved can offer insights into sustaining testing, particularly where test and treat strategies have been maintained post‐elimination [51]. The HIV epidemic in Australia also provides lessons in how to sustain funding for testing in order to reach zero transmission in a context of low incidence. Inner Sydney, Australia was the first locality in the world to reach virtual elimination of HIV transmission [52] and, aside from a reduction in the COVID‐19 era, HIV testing in Australia has stayed relatively stable over the last 10 years [3]. Modelling in Canada indicated that after reaching elimination nationally, moderate levels of testing and treatment coupled with high‐coverage harm reduction programmes would be sufficient to maintain HCV elimination [53].

Applying an overarching, holistic viewpoint to the Building Blocks frameworks allows us to acknowledge the wider structural determinants that impact HCV point‐of‐care testing strategies, such as the conditions of criminalisation, stigma, and discrimination. The context permeates the whole system and is particularly fraught for people who use drugs, who carry a large proportion of HCV infections in Australia [41]. Previous work has highlighted the importance of addressing disease‐related discrimination in order to reorient health systems for people living with HIV [54]. Similarly, the structural factors that allow stigma to proliferate, including racism, sexism and poverty, need to be addressed even if point‐of‐care testing has potential as a tool for stigma reduction by allowing people to test in non‐medical settings. In line with previous research, we must move ‘beyond “test” as “pivotal” for elimination’ [47] and consider the wider context that drives social inequities in health.

This study has some limitations. As previously described, the WHO Health System Building Blocks was not designed for applied research. This study builds on past work that adapts the framework [30], using inductive analysis to generate findings and demonstrating the framework's utility in deriving clear policy implications when investigating complex, interlinked health systems impact. The analysis does not explore the variation in HCV testing programmes across the country but the focus on health systems informs a context to support the implementation of a range of testing strategies. Participants were recruited through the professional networks of the research team that provided access to key informants, but this strategy may have excluded the voices of people in HCV policymaking who are outside that network. Only one participant was recruited from Aboriginal‐led community organisations and as a result, the analysis may not have captured how the health system contributes to social inequities in health experienced by Aboriginal people [55]. This study focused on HCV testing in the community and does not analyse the specific health system challenges faced by HCV testing programmes in the prison. This is a key setting which should be addressed in future health systems research.

The findings from this study have several important policy implications. The identification of multilevel barriers and facilitators in this study, and in other ongoing studies, highlights the need for targeted strategies to optimise the implementation and sustainability of HCV point‐of‐care testing. To ensure long‐term viability, policy efforts should focus on integrating point‐of‐care testing into existing health system structures, securing sustainable funding mechanisms, and strengthening governance frameworks that support quality assurance, data integration and workforce training. Implementation science methodologies offer a systematic approach to addressing these barriers and facilitators [56]. By leveraging these established frameworks and methods, it is possible to circumvent common pitfalls, such as suboptimal resource use, bolster the effect of point‐of‐care testing and accelerate progress towards HCV elimination. Embedding implementation science into policy and practice can help to ensure that innovations in HCV testing lead to equitable and lasting public health improvement.

## Conflicts of Interest

C.T. has received speaker fees from Abbvie and Gilead and has received a research grant from Merck outside the submitted work. J.G. is a consultant/advisor and has received research grants from AbbVie, Abbott, bioLytical, Cepheid, Gilead Sciences, Hologic and Roche outside the submitted work. L.L. has received speaker fees from AbbVie. G.F. has received speaker fees/hospitality from Abbvie and Gilead. A.D.M. is currently a CI on a Gilead research grant. A.C. and S.J.M. declare that they have no conflicts of interest.

## Data Availability

Research data are not shared.

## References

[1] E. Ogawa , N. Chien , L. Kam , et al., “Association of Direct‐Acting Antiviral Therapy With Liver and Nonliver Complications and Long‐Term Mortality in Patients With Chronic Hepatitis C,” JAMA Internal Medicine 183, no. 2 (2023): 97–105, 10.1001/jamainternmed.2022.5699. 36508196 PMC9856614

[2] Burnet Institute and Kirby Institute , “Australia’s progress Towards Hepatitis C Elimination: Annual Report 2023,” (2023), Melbourne: Burnet Institute, 10.26190/4D4F-5N41.

[3] J. King , H. McManus , J. Kwon , R. Gray , and S. McGregor , “HIV, Viral Hepatitis and Sexually Transmissible Infections in Australia: Annual Surveillance Report 2023,” (2023), Kirby Institute, UNSW Sydney, Sydney, UNSW, 10.26190/F5PH-F972.

[4] World Health Organization , “Implementing the Global Health Sector Strategies on HIV, Viral Hepatitis and Sexually Transmitted Infections, 2022–2030: Report on Progress and Gaps 2024, Second Edition,” (2024).

[5] J. Grebely , T. L. Applegate , P. Cunningham , and J. J. Feld , “Hepatitis C Point‐of‐Care Diagnostics: In Search of a Single Visit Diagnosis,” Expert Review of Molecular Diagnostics 17, no. 12 (2017): 1109–1115, 10.1080/14737159.2017.1400385. 29088981

[6] J. Grebely , S. Matthews , L. M. Causer , et al., “We Have Reached Single‐Visit Testing, Diagnosis, and Treatment for Hepatitis C Infection, Now What?,” Expert Review of Molecular Diagnostics 24 (2024): 177–191, 10.1080/14737159.2023.2292645. 38173401

[7] E. Cunningham , A. Wheeler , B. Hajarizadeh , et al., “Interventions to Enhance Testing and Linkage to Treatment for Hepatitis C Infection: A Systematic Review and Meta‐Analysis,” Lancet Gastroenterology & Hepatology 111 (2023): 103917. 10.1016/S2468-1253(21)00471-4 35303490

[8] J. Grebely , R. Gilliver , T. McNaughton , et al., “Single‐Visit Hepatitis C Point‐of‐Care Testing, Linkage to Nursing Care, and Peer‐Supported Treatment Among People With Recent Injecting Drug Use at a Peer‐Led Needle and Syringe Program: The TEMPO Pilot Study,” International Journal of Drug Policy 114 (2023): 103982, 10.1016/j.drugpo.2023.103982. 36863287

[9] A. Trickey , E. Fajardo , D. Alemu , A. A. Artenie , and P. Easterbrook , “Impact of Hepatitis C Virus Point‐of‐Care RNA Viral Load Testing Compared With Laboratory‐Based Testing on Uptake of RNA Testing and Treatment, and Turnaround Times: A Systematic Review and Meta‐Analysis,” Lancet Gastroenterology & Hepatology 8, no. 3 (2023): 253–270, 10.1016/S2468-1253(22)00346-6. 36706775 PMC11810864

[10] A. Conway , H. Valerio , M. Alavi , et al., “A Testing Campaign Intervention Consisting of Peer‐Facilitated Engagement, Point‐of‐Care HCV RNA Testing, and Linkage to Nursing Support to Enhance Hepatitis C Treatment Uptake Among People Who Inject Drugs: The ETHOS Engage Study,” Viruses 14, no. 7 (2022): 1555, 10.3390/v14071555. 35891535 PMC9316739

[11] X. Forns , J. Colom , M. García‐Retortillo , et al., “Point‐of‐Care Hepatitis C Testing and Treatment Strategy for People Attending Harm Reduction and Addiction Centres for Hepatitis C Elimination,” Journal of Viral Hepatitis 29, no. 3 (2022): 227–230, 10.1111/jvh.13634. 34806812 PMC9299793

[12] A. Conway , A. Stevens , C. Murray , et al., “Hepatitis C Treatment Uptake Following Dried Blood Spot Testing for Hepatitis C RNA in New South Wales, Australia: The NSW DBS Pilot Study,” Open Forum Infectious Diseases 10, no. 11 (2023): ofad517, 10.1093/ofid/ofad517. 38023551 PMC10665037

[13] E. B. Cunningham , A. Wheeler , B. Hajarizadeh , et al., “Interventions to Enhance Testing and Linkage to Treatment for Hepatitis C Infection for People Who Inject Drugs: A Systematic Review and Meta‐Analysis,” International Journal of Drug Policy 111 (2023): 103917, 10.1016/j.drugpo.2022.103917. 36542883

[14] E. Day , M. Hellard , C. Treloar , et al., “Hepatitis C Elimination Among People Who Inject Drugs: Challenges and Recommendations for Action Within a Health Systems Framework,” Liver International 39, no. 1 (2019): 20–30, 10.1111/liv.13949. 30157316 PMC6868526

[15] World Health Organization , “Updated Recommendations on Treatment of Adolescents and Children With Chronic HCV Infection, and HCV Simplified Service Delivery and Diagnostics,” (2022). 37410875

[16] K. J. Land , D. I. Boeras , X. S. Chen , A. R. Ramsay , and R. W. Peeling , “REASSURED Diagnostics to Inform Disease Control Strategies, Strengthen Health Systems and Improve Patient Outcomes,” Nature Microbiology 4, no. 1 (2018): 46–54, 10.1038/s41564-018-0295-3. PMC7097043 30546093

[17] S. T. F. Shih , Q. Cheng , J. Carson , et al., “Optimizing Point‐of‐Care Testing Strategies for Diagnosis and Treatment of Hepatitis C Virus Infection in Australia: A Model‐Based Cost‐Effectiveness Analysis,” Lancet Regional Health – Western Pacific 36 (2023): 100750, 10.1016/j.lanwpc.2023.100750. 37547040 PMC10398594

[18] J. Grebely , “Point‐of‐Care Testing for Hepatitis C Infection: A Critical Building Block for the Foundation to Achieve Elimination,” Clinical Infectious Diseases 79, no. 4 (2024): 974–977, 10.1093/cid/ciae156. 38513081

[19] J. E. Moore , A. Mascarenhas , J. Bain , and S. E. Straus , “Developing a Comprehensive Definition of Sustainability,” Implementation Science 12, no. 1 (2017): 110, 10.1186/s13012-017-0637-1. 28865479 PMC5581411

[20] J. Grebely , C. Markus , L. M. Causer , et al., “A National Programme to Scale‐Up Decentralised Hepatitis C Point‐of‐Care Testing and Treatment in Australia,” Lancet Gastroenterology & Hepatology 8, no. 3 (2023): 204–207, 10.1016/S2468-1253(22)00355-7. 36773609

[21] Therapeutic Goods Administration, Commonwealth of Australia , “Classification of IVD Medical Devices,” (2020).

[22] National Pathology Accreditation Advisory Council (NPAAC) , “Requirements for Point of Care Testing (Second Edition 2021),” Commonwealth of Australia as represented by the Department of Health, 2021, (2021), https://www.safetyandquality.gov.au/sites/default/files/2022‐08/tier_4_requirements_for_point_of_care_testing_second_edition_2021.pdf.

[23] Australian Government – Department of Health and Aged Care , “Medicare Benefits Schedule – Item 69499,” (2025), accessed January 8, 2025, https://www9.health.gov.au/mbs/fullDisplay.cfm?type=item&q=69499&qt=ItemID.

[24] World Health Organization , “Everybody's Business—Strengthening Health Systems to Improve Health Outcomes: WHO's Framework for Action,” (2007): p. 44.

[25] P. Hanvoravongchai , S. Mounier‐Jack , V. Oliveira Cruz , et al., “Impact of Measles Elimination Activities on Immunization Services and Health Systems: Findings From Six Countries,” Journal of Infectious Diseases 204, no. suppl_1 (2011): S82–S89, 10.1093/infdis/jir091. 21666218

[26] D. Yu , Y. Souteyrand , M. A. Banda , J. Kaufman , and J. H. Perriëns , “Investment in HIV/AIDS Programs: Does It Help Strengthen Health Systems in Developing Countries?,” Globalization and Health 4, no. 1 (2008): 8, 10.1186/1744-8603-4-8. 18796148 PMC2556650

[27] H. Amu , R. K. Dowou , F. I. Saah , J. A. Efunwole , L. E. Bain , and E. E. Tarkang , “COVID‐19 and Health Systems Functioning in Sub‐Saharan Africa Using the “WHO Building Blocks”: The Challenges and Responses,” Frontiers in Public Health 10 (2022): 856397, 10.3389/fpubh.2022.856397. 35444973 PMC9013894

[28] J. Borghi and G. W. Brown , “Taking Systems Thinking to the Global Level: Using the WHO Building Blocks to Describe and Appraise the Global Health System in Relation to COVID‐19,” Global Policy 13, no. 2 (2022): 193–207, 10.1111/1758-5899.13081. 35601655 PMC9111126

[29] E. Day , S. Matthews , C. Markus , et al., “Barriers and Solutions to Increasing Access to Point‐of Care HCV Testing: Recommendations From the 2023 INHSU Hepatitis C Point‐of‐Care Testing Forum,” INHSU, (2023).

[30] S. Mounier‐Jack , U. K. Griffiths , S. Closser , H. Burchett , and B. Marchal , “Measuring the Health Systems Impact of Disease Control Programmes: A Critical Reflection on the WHO Building Blocks Framework,” BMC Public Health 14, no. 1 (2014): 278, 10.1186/1471-2458-14-278. 24666579 PMC3974593

[31] E. Sacks , M. Morrow , W. T. Story , et al., “Beyond the Building Blocks: Integrating Community Roles Into Health Systems Frameworks to Achieve Health for All,” BMJ Global Health 3, no. Suppl 3 (2019): e001384, 10.1136/bmjgh-2018-001384. PMC6591791 31297243

[32] R. J. Guy , J. Ward , L. M. Causer , et al., “Molecular Point‐of‐Care Testing for Chlamydia and Gonorrhoea in Indigenous Australians Attending Remote Primary Health Services (TTANGO): A Cluster‐Randomised, Controlled, Crossover Trial,” Lancet Infectious Diseases 18, no. 10 (2018): 1117–1126, 10.1016/S1473-3099(18)30429-8. 30303108

[33] N. Crofts and D. Herkt , “A History of Peer‐Based Drug‐User Groups in Australia,” Journal of Drug Issues 25, no. 3 (1995): 599–616, 10.1177/002204269502500306.

[34] C. Henderson , A. Madden , and J. Kelsall , “‘Beyond the Willing & the Waiting’—The Role of Peer‐Based Approaches in Hepatitis C Diagnosis & Treatment,” International Journal of Drug Policy 50 (2017): 111–115, 10.1016/j.drugpo.2017.08.004. 28927831

[35] A. D. Marshall , M. Hopwood , J. Grebely , and C. Treloar , “Applying a Diffusion of Innovations Framework to the Scale‐Up of Direct‐Acting Antiviral Therapies for Hepatitis C Virus Infection: Identified Challenges for Widespread Implementation,” International Journal of Drug Policy 86 (2020): 102964, 10.1016/j.drugpo.2020.102964. 33059118

[36] A. J. Milat , L. King , R. Newson , et al., “Increasing the Scale and Adoption of Population Health Interventions: Experiences and Perspectives of Policy Makers, Practitioners, and Researchers,” Health Research Policy and Systems 12, no. 1 (2014): 18, 10.1186/1478-4505-12-18. 24735455 PMC3996855

[37] NSW Health , “HIV and Hepatitis C Dried Blood Spot (DBS) Test,” (2024), accessed October 31, 2024, https://www.health.nsw.gov.au/dbstest/Pages/default.aspx.

[38] B. Hengel , R. J. Guy , D. Casey , et al., “Decentralised COVID‐19 Molecular Point‐of‐Care Testing: Lessons From Implementing a Primary Care‐Based Network in Remote Australian Communities,” Medical Journal of Australia 222, no. 4 (2025): 172–178, 10.5694/mja2.52589. 39888019

[39] D. J. Cohen , B. F. Crabtree , R. S. Etz , et al., “Fidelity Versus Flexibility,” American Journal of Preventive Medicine 35, no. 5 (2008): S381–S389, 10.1016/j.amepre.2008.08.005. 18929985

[40] The Kirby Institute , “Federal Budget Allocation Will Expand Access to STI Testing for Thousands in Remote Communities,” accessed February 7, 2024, https://www.kirby.unsw.edu.au/news/federal‐budget‐allocation‐will‐expand‐access‐sti‐testing‐thousands‐remote‐communities.

[41] A. Trickey , H. Fraser , A. G. Lim , et al., “The Contribution of Injection Drug Use to Hepatitis C Virus Transmission Globally, Regionally, and at Country Level: A Modelling Study,” Lancet Gastroenterology & Hepatology 4, no. 6 (2019): 435–444, 10.1016/S2468-1253(19)30085-8. 30981685 PMC6698583

[42] C. Treloar , J. Rance , and M. Backmund , “Understanding Barriers to Hepatitis C Virus Care and Stigmatization From a Social Perspective,” Clinical Infectious Diseases 57, no. Suppl 2 (2013): 51–55, 10.1093/cid/cit263. 23884066

[43] S. Crawford and N. Bath , “Peer Support Models for People With a History of Injecting Drug Use Undertaking Assessment and Treatment for Hepatitis C Virus Infection,” Clinical Infectious Diseases 57, no. Suppl_2 (2013): S75–S79, 10.1093/cid/cit297. 23884070

[44] W. Dimech , L. Cabuang , K. Davies , and G. Vincini , “Implementation of Novel Quality Assurance Program for Hepatitis C Viral Load Point of Care Testing,” Viruses 14, no. 9 (2022): 1929, 10.3390/v14091929. 36146736 PMC9504144

[45] L. Lafferty , T. L. Applegate , S. Lewis , et al., “Pre‐Market Health Systems Barriers and Enablers to Infectious Diseases Point‐of‐Care Diagnostics in Australia: Qualitative Interviews With Key Informants,” BMC Infectious Diseases 24, no. 1 (2024): 1317, 10.1186/s12879-024-10214-5. 39558237 PMC11575463

[46] A. Saha , K. Andrewartha , S. G. Badman , et al., “Flexible and Innovative Connectivity Solution to Support National Decentralized Infectious Diseases Point‐of‐Care Testing Programs in Primary Health Services: Descriptive Evaluation Study,” Journal of Medical Internet Research 25 (2023): e46701, 10.2196/46701. 37656506 PMC10504621

[47] C. Treloar , J. Rance , J. Bryant , and L. Lafferty , “‘There's Too Much Power in This Number. It's Freaking the Whole Response out’: The Views of Key Informants on Evidence and Targets to Achieve Hepatitis C Elimination Goals in Australia,” Journal of Viral Hepatitis 31, no. 2 (2024): 59–65, 10.1111/jvh.13896. 37916576

[48] M. Harris , T. Rhodes , and A. Martin , “Taming Systems to Create Enabling Environments for HCV Treatment: Negotiating Trust in the Drug and Alcohol Setting,” Social Science and Medicine 83 (2013): 19–26, 10.1016/j.socscimed.2013.01.031. 23465200

[49] Australian Government Department of Health and Aged Care , “Sixth National Hepatitis C Strategy 2023–2030,” Department of Health and Aged Care Australia, (2023).

[50] NSW Health , “NSW Hepatitis C Strategy 2022‐2025,” NSW Health, (2022), accessed March 5, 2024, https://www.health.nsw.gov.au/hepatitis/Pages/hepatitiscstrategy.aspx.

[51] A. Cuadrado , S. Llerena , C. Cobo , et al., “Microenvironment Eradication of Hepatitis C: A Novel Treatment Paradigm,” American Journal of Gastroenterology 113, no. 11 (2018): 1639–1648, 10.1038/s41395-018-0157-x. 29946175

[52] International AIDS Society , “HIV Transmission Virtually Eliminated in Inner Sydney, Australia,” (2023), accessed July 24, 2023, https://www.iasociety.org/news‐release/hiv‐transmission‐virtually‐eliminated‐inner‐sydney‐australia.

[53] C. L. Delaunay , A. Godin , N. Kronfli , et al., “Can Hepatitis C Elimination Targets Be Sustained Among People Who Inject Drugs Post‐2030?,” International Journal of Drug Policy 96 (2021): 103343, 10.1016/j.drugpo.2021.103343. 34215459

[54] K. Safreed‐Harmon , J. Anderson , N. Azzopardi‐Muscat , et al., “Reorienting Health Systems to Care for People With HIV Beyond Viral Suppression,” Lancet HIV 6, no. 12 (2019): e869–e877, 10.1016/S2352-3018(19)30334-0. 31776099

[55] C. Treloar , L. C. Jackson , R. Gray , et al., “Multiple Stigmas, Shame and Historical Trauma Compound the Experience of Aboriginal Australians Living With Hepatitis C,” Health Sociology Review 25, no. 1 (2016): 18–32, 10.1080/14461242.2015.1126187.

[56] G. Fontaine , N. Taylor , J. Bruneau , et al., “The Urgent Need for Implementation Science to Achieve Hepatitis C Elimination,” Lancet Gastroenterology & Hepatology 10 (2025): S2468125325000500, 10.1016/S2468-1253(25)00050-0. 40054488

